# Factors Associated with Defecation Satisfaction among Japanese Adults with Chronic Constipation

**DOI:** 10.3390/jcm13113216

**Published:** 2024-05-30

**Authors:** Sayuri Yamamoto, Wataru Ohashi, Yoshiharu Yamaguchi, Hiroki Igari, Akira Koshino, Tomoya Sugiyama, Kazuhiro Nagao, Yasuhiro Tamura, Shinya Izawa, Mamiko Mano, Masahide Ebi, Jun Usami, Koichi Hamano, Junko Izumi, Yoshinori Wakita, Yasushi Funaki, Naotaka Ogasawara, Ryujiro Sasanabe, Makoto Sasaki, Masato Maekawa, Kunio Kasugai

**Affiliations:** 1 Division of Gastroenterology, Aichi Medical University, Nagakute 480-1195, Japan; 2 Division of General Medicine, Aichi Medical University, Nagakute 480-1195, Japan; 3 Division of Biostatistics, Clinical Research Center, Aichi Medical University, Nagakute 480-1195, Japan; 4 Division of Sleep Medicine, Aichi Medical University, Nagakute 480-1195, Japan

**Keywords:** constipation, internet survey, laxative, treatment satisfaction

## Abstract

**Background:** Constipation causes substantial morbidity worldwide. **Methods:** This survey assessed constipation-related factors in Japan using the Japanese version of the Irritable Bowel Syndrome Quality of Life (IBS-QOL-J) instrument. We also examined the relationship among laxative type, Bristol Stool Form Scale (BSFS) scores, and treatment cost. Finally, we examined differences in satisfaction scores according to laxative type, treatment type, treatment cost, and BSFS score. **Results:** IBS-QOL-J was higher among those taking salt and/or irritation laxatives. Those paying >JPY 5000 (USD 50.00) had the lowest IBS-QOL-J. IBS-QOL-J was significantly lower among those with a BSFS score of 1 or 2 (severe constipation). **Conclusions:** This study’s findings suggest that a variety of factors, including treatment type and cost, are associated with defecation satisfaction. Those who had hard stools, used multiple laxatives, or spent more on treatment were less satisfied. Future strategies should target therapies that do not require multiple laxatives with lower treatment costs. Adequate defecation with a small number of appropriate laxatives at minimal cost appears to improve defecation satisfaction. It is desirable to identify appropriate laxatives and improve dietary habits and exercise routines. It is also necessary to stop blindly increasing laxative usage and properly diagnose constipation disorders such as anatomical abnormalities other than functional constipation.

## 1. Introduction

Constipation is a common gastrointestinal disorder that results in altered bowel movements. This condition could either be a primary disorder or a secondary issue that arises as a consequence of another underlying disease, such as cancer, or due to medication [1]. Constipation is classically defined as a decrease in the frequency of stools as well as a reduction in the ease of the passage of stools compared to normal stool passage patterns [2]. Individuals suffering from constipation very often feel bloated and have hard stools and a sensation of incomplete evacuation [3]. All individuals experience constipation during some period of their lives due to altered dietary or lifestyle conditions. While acute constipation is typically thought to be a benign condition, chronic constipation can have a negative impact on sleep, mental health, and quality of life as well as impose a significant economic burden on the individual [4,5,6,7,8,9]. Studies have also shown associations between chronic constipation and adverse clinical outcomes such as cardiovascular disease, stroke, and mortality [10,11]. In Japan, chronic constipation has been shown to adversely affect productivity at work as well as health-related quality of life and is reported to be worse than other diseases such as type 2 diabetes mellitus, gastroesophageal reflux disease, and irritable bowel syndrome [12]. 

The underlying causes of constipation have been attributed to a variety of factors, such as the type and quantity of food and fluid intake, lifestyle factors, genetic predisposition, level of physical activity, medications, hormones, etc. [13,14,15]. This diversity in the causes of constipation, along with how constipation is measured, has resulted in a wide range in the prevalence of constipation, which has been reported to be 2 to 27% worldwide [16]. Prevalence of constipation can also vary between countries, potentially due to a variety of cultural, genetic, and environmental factors [17]. An internet survey conducted in Japan estimated the prevalence of constipation at 28.4%, with constipation being more common among females than males [18]. Another survey conducted among individuals younger than 70 years in Japan found that the prevalence of functional constipation was 9% and that of constipation-predominant irritable bowel syndrome was 5% [19].

There is no ideal or clear definition of constipation. It is typically diagnosed using self-reported history of bowel functions, physical examination, and formal criteria [20,21]. The two formal criteria for diagnosing constipation are either the American Gastroenterological Association (AGA) criteria or the Rome criteria [21]. The AGA criteria analyze colonic transit as well as anorectal tests to diagnose and classify constipation into different groups [22]. The Rome criteria (the latest version is the Rome IV criteria) consist of a questionnaire that is extensively used to diagnose functional gastrointestinal disorders [23,24]. Using the Rome criteria, cases of constipation are separated into different severity levels according to a variety of factors, including the duration of constipation, the amount of straining individuals experience while passing stool, as well as stool consistency. The Bristol Stool Scale (BSFS) is a tool often used to assess stool consistency. It categorizes feces into seven different types according to consistency. While Types 3 and 4 on this scale are normal stools, Types 1 and 2 are very hard stools and thus indicate severe constipation [23].

There are many therapies available for the treatment of constipation. Treatment strategies are primarily aimed at increasing fiber intake and the use of suppositories, enemas [1], as well as irritant and osmotic laxatives [25]. A previous meta-analysis identified laxatives as an effective treatment for constipation [26]. In cases of pelvic floor dyssynergia, biofeedback training appears to improve bowel function and constipation [27]. In rare cases of very severe constipation involving pelvic floor coordination disorder and reduced abdominal pressure, among other symptoms, surgical treatment may be necessary [28]. There are several home remedies, natural foods, over-the-counter therapies, as well as prescribed medications available for treating constipation. However, there are a variety of advantages and disadvantages to these different treatment methods, and some treatments may be more effective for some patients than others [29,30,31,32]. The course or type of treatment used often depends on the severity of the symptoms; however, the cost of these treatments could also contribute to the treatment course [33]. The effectiveness of treatment strategies for constipation has been largely inconclusive due to the differences in opinion between the patients and their treating physicians [34]. One study suggested that physicians may be more prone to focus only on stool frequency, and the same treatment may not necessarily lead to defecation satisfaction for the patient [35]. While the treating physicians tend to objectively evaluate the treatment by examination of stool form and the frequency of bowel movements, patient satisfaction with the treatment also includes subjective evaluation, such as a sense of complete evacuation as abdominal bloating, etc. [36]. Indeed, a multicenter study in Japan showed that patients treated for chronic constipation were not satisfied with their treatment even though they were classified as having a stool form of BSFS Type 3, which is considered normal [37]. Additionally, the medications used to treat constipation in Japan vary from those in other countries due to availability, approvals, and/or cost [38].

The problem of constipation and its treatment can vary widely across individuals across different countries and ethnicities. Treatment of constipation requires treatment of the symptoms as well as improving the quality of life of individuals with constipation. From the perspective of patients, quality of life is significantly influenced by a sense of having a satisfactory bowel movement with complete evacuation. We thus sought to determine whether defecation satisfaction, as measured by satisfaction using the Irritable Bowel Syndrome Quality of Life Japanese version (IBS-QOL-J) scores, differed according to the type of drug used, the treatment type, the cost of treatment, and the BSFS score in Japan.

## 2. Methods

This study used data collected by a survey research company, Rakuten Insight, located in Osaka City. The data were collected on 8–11 October 2016, and a total of 9523 individuals were surveyed. This survey (Supplementary Table S1) was conducted on a randomized sample of individuals who were enrolled in the survey company’s monitors and who were matched to the age gender demographic composition of each prefecture population in Japan. All 47 prefectures were represented in this study. The survey was aimed at individuals who thought they suffered from chronic constipation. Of the surveyed individuals, 4615 respondents reported that they were of the opinion that they suffered from constipation. From this group of those who were aware of their constipation, 3000 were randomly selected, as described previously, to be representative of the Japanese population by prefecture [5,39]. Those with secondary constipation due to medication or those suffering from other diseases such as cancer or neuroendocrine disorders were excluded, as mentioned previously [5,39].

The specific variables collected for this study included the BSFS to assess consistency of the stools [19], the IBS-QOL-J to assess patient defecation satisfaction [19], whether medication was taken for constipation, and the type of medication taken. 

The BSFS scale consists of seven types of feces based on consistency and is used to determine the severity of constipation [19]. For the purpose of this study, we grouped Types 1 and 2 together to indicate hard stool, Types 3–5 to indicate normal feces, and Types 6 and 7 to indicate diarrhea. 

The IBS-QOL-J was used to assess defecation satisfaction, as described previously [19]. This survey has been used previously in Japan to effectively evaluate the quality of life of individuals with functional constipation or constipation-predominant IBS [19]. The IBS-QOL-J has been translated and validated previously from the IBS-QOL questionnaire that is used widely in Western countries [40]. The Japanese version of the IBS-QOL has 38 items in several categories, such as worry of food, social reactions, dysphoria, etc., and these are scored on a scale of 0 to 4, with 0 indicating absent/no and 4 indicating very strongly. Higher IBS-QOL-J scores indicated greater defecation satisfaction. The average treatment cost of all medications used for constipation was collected based on the self-reported responses on the questionnaire and used to determine the average cost in one month. These data were divided into 4 groups for the purpose of analyzing defecation satisfaction based on treatment cost. Finally, demographic variables, including age and sex, were collected. 

The patients were grouped based on the kind of medication that they were taking for constipation. The different types of medications used were irritant laxatives (such as aloe, phenolphthalein series, Pruzenid, etc.), enema suppositories (including rectal suppositories and/or enemas), or osmotic laxatives (such as the saline laxative magnesium oxide). Cases where the laxative used was unknown constituted a separate group. To examine defecation satisfaction according to how the medication was purchased, patients were classified into 3 groups: whether the medication was purchased from a pharmacy, online, or following a doctor’s prescription. Defecation satisfaction was also examined according to the average cost of treatment per month.

Data are presented as mean ± standard deviation (SD) or median (interquartile range [IQR]). The Mann–Whitney U test or the Kruskal–Wallis test was used for intergroup comparisons of numerical or ordinal scale data, as appropriate. The Bonferroni correction was applied as necessary. The chi-square test or residual test was used for intergroup comparisons of categorical data. The Mann–Whitney U test was used for comparisons between gender and IBS-QOL-J scores. Pearson’s correlation was used to test the relation between age and IBS-QOL-J scores, and Spearman’s rank correlation was used to test the relation between annual income or education history and IBS-QOL-J scores. The level of significance was set to 0.05 (two-sided) for all the analyses. Holm’s method was used to correct for test multiplicity. SAS ver. 9.4 for Windows (SAS Institute Japan LTD., Tokyo, Japan) was used for all statistical analysis. 

The study was approved by the Aichi Medical University Ethics Review Committee (no. 2016-M025). This research was conducted in accordance with the Declaration of Helsinki of the World Medical Association and the ethical guidelines for human medical research by the Ministry of Education, Culture, Sports, Science, and Technology and the Ministry of Health, Labour and Welfare (established 22 December 2014). The statistical analysis in this research was done by our staff and confirmed by Mr. Wataru Ohashi, Associate Professor at the Clinical Research Support Centre of Aichi Medical University. Informed consent was obtained from all study participants. 

## 3. Results

A total of 3000 (1503 males, 1497 females; mean age, 43.1 ± 14.7 years) randomly selected individuals who indicated that they suffered from constipation participated in this study. These 3000 participants were selected as described previously [12] to be representative of the Japanese population in age and gender by prefecture. The demographics of the study population are shown in Table 1. 

Constipation is known to be a heterogenous condition that may be influenced by a variety of factors such as age, sex, socioeconomic status, etc. Unfortunately, as the data for this study were based on ordinal scales, we were not able to perform multivariate analysis for various factors. We did, however, perform stratified analysis for defecation satisfaction scores for basic background factors. There was no significant difference between gender and IBS-QOL-J scores (*p* = 0.277) and no correlation between age and IBS-QOL-J scores (Pearson’s correlation coefficient, r = 0.177). Similarly, there was no correlation between annual income and IBS-QOL-J scores (Spearman’s rank correlation coefficient, ρ = 0.0264) and between education history and IBS-QOL-J scores (ρ = 0.0186) (Supplementary Table S2). 

**Figure 1:**
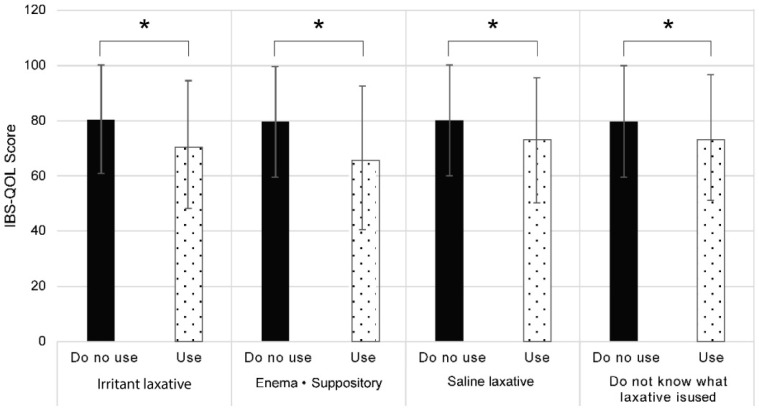
Japanese version of the Irritable Bowel Syndrome Quality of Life (IBS-QOL-J) score for different laxatives used. Bar graphs of the IBS-QOL-J score for individuals using a particular laxative or not are shown. The results are expressed as mean ± standard deviation. * *p* < 0.001 indicates significantly different from non-usage (Mann–Whitney U test).

We examined defecation satisfaction for individuals who used a particular laxative compared to those who were not using any medication. Figure 1 shows a comparison of IBS-QOL-J scores for individuals who used a particular laxative with those who did not. The mean and median scores were significantly lower among those who used laxatives compared to those who did not. The type of laxative did not affect this decrease in the IBS-QOL-J scores. The mean and median IBS-QOL-J scores were also significantly lower for individuals who did not know the laxative that was used compared to those who used no laxatives.

**Figure 2:**
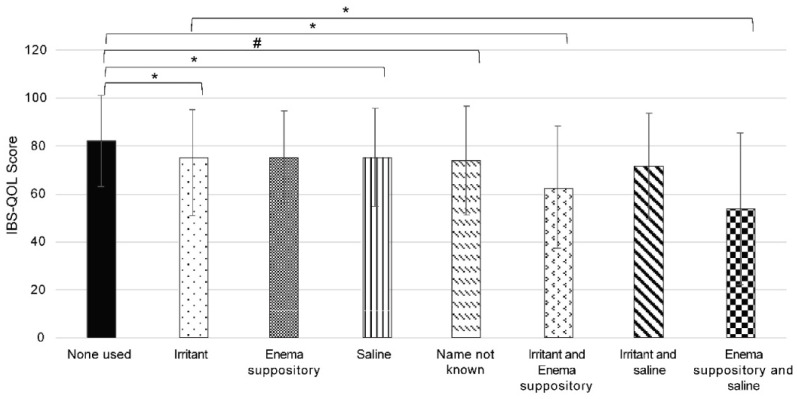
Japanese version of the Irritable Bowel Syndrome Quality of Life (IBS-QOL-J) score according to the laxative or laxative combinations used. Bar graphs of the IBS-QOL-J scores for individuals using one laxative or a combination of laxatives are shown. The results are expressed as the mean ± standard deviation. * *p* < 0.001 and # *p* < 0.05 indicate a statistically significant difference using the Mann–Whitney U test followed by Bonferroni correction.

We next examined whether there were any differences in defecation satisfaction among individuals using different types of laxatives. As shown in Figure 2, there were differences in the mean and median IBS-QOL-J scores among individuals using different laxatives. Those who did not use laxatives had significantly higher scores than those who used any suppositories or combinations thereof, except for enema suppositories. There were some individuals in the population who used combinations of different laxatives. Among the individuals who used combination laxatives, the combination of enema suppositories and saline had significantly lower scores than all other single laxatives or combinations, with the exception of the combination of irritant and enema suppositories.

**Figure 3:**
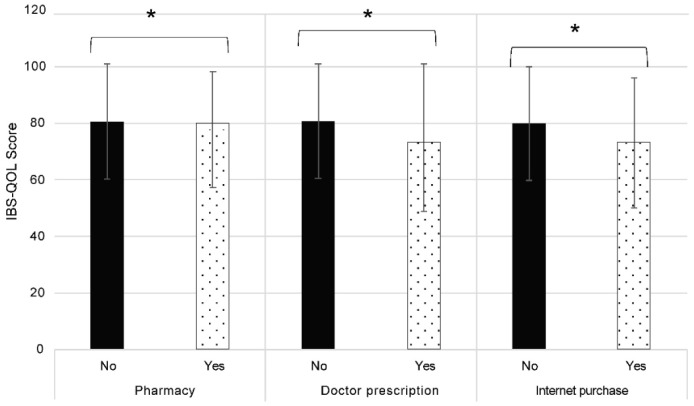
Japanese version of the Irritable Bowel Syndrome Quality of Life (IBS-QOL-J) score according to mode of purchase. Bar graphs of the IBS-QOL-J score depending on how the medication was purchased are shown. The results are expressed as mean ± standard deviation. Statistical significance was determined at * *p* < 0.0001 using the Mann–Whitney U test.

This study consisted of individuals with self-reported constipation and hence included those who were self-medicating as well as those who were taking medications prescribed by a physician. Medication for treating constipation in Japan can either be purchased at a pharmacy, online, or following a doctor’s prescription. We examined differences in defecation satisfaction among individuals based on how the medication was purchased. Whether the medication was purchased at a hospital following a doctor’s prescription or from a pharmacy or the internet resulted in a small but significant decrease in defecation satisfaction, as shown in Figure 3. These results suggest that defecation satisfaction was independent of how the medication was procured.

The cost of treatment for constipation can vary greatly due to improper self-diagnosis, self-medication, and use of home remedies or over-the-counter medication that may not always be effective [41]. We examined defecation satisfaction in individuals according to the average cost of treatment per month. 

**Figure 4:**
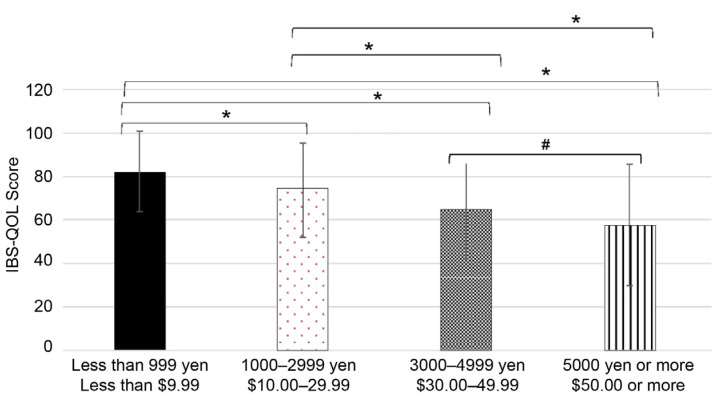
Japanese version of the Irritable Bowel Syndrome Quality of Life (IBS-QOL-J) score according to treatment cost per month expressed in JPY and USD. Bar graphs of the IBS-QOL-J score depending on treatment cost are shown. The results are expressed as mean ± standard deviation. * *p* < 0.001 and # *p* < 0.05 indicate a statistically significant difference using the Mann–Whitney U test followed by Bonferroni correction.

As shown in Figure 4, satisfaction scores varied according to treatment cost. Interestingly, defecation satisfaction decreased with increasing cost. In fact, individuals who spent less than JPY 999 (USD 9.99) had the highest IBS-QOL-J score. 

**Figure 5:**
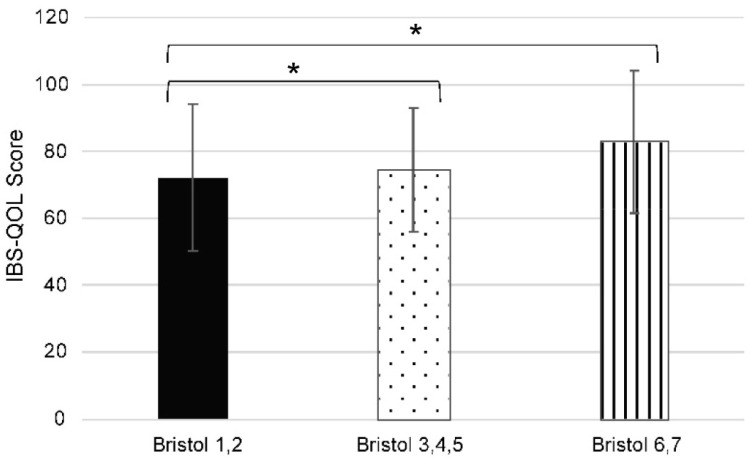
Japanese version of the Irritable Bowel Syndrome Quality of Life (IBS-QOL-J) score according to the Bristol Stool Form Score (BSFS). Bar graphs of the IBS-QOL-J score depending on the BSFS score are shown. The BSFS was grouped into 3 groups, with Type 1 and 2 indicating severe constipation, Types 3–5 indicating normal stools, and Types 6 and 7 indicating diarrhea. The results are expressed as mean ± standard deviation. Significance was determined at * *p* < 0.001 using the Kruskal–Wallis test followed by Bonferroni correction.

Stool frequency and stool form or consistency are important in determining treatment efficacy. In order to better understand the contribution of stool form to defecation satisfaction, we examined IBS-QOL-J scores depending on the consistency of the stools as evaluated by BSFS. Defecation satisfaction differed significantly depending on BSFS. As expected, those with BSFS scores of 1 and 2 (which is indicative of severe constipation) had significantly lower IBS-QOL-J scores than those with BSFS scores of ≥3 (Figure 5).

## 4. Discussion

In this study, we examined defecation satisfaction in individuals with chronic constipation using a Japanese version of the Quality of Life questionnaire. Ironically, we found that individuals who used laxatives had significantly lower defecation satisfaction. Defecation satisfaction scores were also significantly lower among those paying more for treatment, with those spending less than JPY 999 (USD 9.99) having significantly higher defecation satisfaction. BSFS was also associated with defecation satisfaction, and as expected, individuals with a lower BSFS tended to have lower IBS-QOL scores. 

In our study, only severely constipated individuals (with BSFS scores of 1 and 2) had significantly lower IBS-QOL-J scores. Individuals with high BSFS scores (that would indicate diarrhea) had IBS-QOL-J scores similar to those having normal stools. Previous studies have shown that individuals with a BSFS score of 4 (normal stool) have the highest QOL [35]. Taken together, these results suggest that improving stool quality may be an effective method for improving defecation satisfaction. 

Treatment of chronic constipation can involve a variety of prescribed pharmacological agents, over-the-counter drugs, as well as natural food products. While the first line of treatment for constipation typically involves improving lifestyle habits such as diet and exercise, there have been recent published guidelines to provide a framework that would help in the treatment and care of patients with chronic constipation [42]. Previous research has suggested that laxative usage is an effective means of treating constipation [26,33]. However, laxatives typically do not require a prescription from a doctor and hence are indiscriminately used to self-medicate in the case of constipation. This often results in the treatment being ineffective, either due to inaccurate dosage or improper choice of the laxative used [43]. Indeed, previous studies have shown that some treatments may result in improvement in some symptoms, such as stool form, while exacerbating others, such as bloating [44]. These worsened symptoms may in turn affect individuals’ QOL [45]. The results of our study suggest that laxative usage does not improve QOL. In contrast, QOL appears to be lower in individuals taking laxatives. It is possible that in our study, these results may also be confounded by the degree of constipation in each individual or other confounding factors that were not examined in this study. In our study, there were 2074 (69.1%) individuals who were not currently taking any medication. Since all the data in this study were self-reported or from self-evaluated constipation, some individuals may not have felt the need to take laxatives for their constipation. It is also possible that some of these individuals were making lifestyle changes to treat their symptoms. Indeed, a survey of American individuals experiencing constipation found that around 28% of individuals were not taking any medication for their constipation [45]. Previous research in Japan reported that only 30% of surveyed individuals with self-reported constipation used laxatives to treat their constipation [46]. Further research examining this in a longitudinal fashion may provide more insight into this issue. 

Pharmacological drugs to treat constipation in Japan can be purchased online, at a pharmacy, or following a doctor’s prescription. Drugs that require a prescription are purchased from prescription-only pharmacies, while drugs that do not require prescriptions can be purchased online or over-the-counter from pharmacies. A study in Japan examining the influence of consultations with a pharmacist on the awareness and purchase of non-prescription laxatives found that patients who consulted pharmacists had greater awareness and used laxatives more appropriately than those who did not. The study found that while patients with constipation often self-medicate, many consult a pharmacist and make lifestyle changes to treat their constipation prior to taking laxatives, if required [47]. In this study, however, we found that defecation satisfaction was independent of how the medication was procured.

Constipation as well as details about the type of laxatives taken were self-reported by the participating individuals. Our results show that 26.4% of individuals classified as Bristol 3, 4, 5, and 35.2% of individuals classified as Bristol 6,7 were taking laxatives. It remains possible that these individuals were taking medications that resulted in a change of their Bristol scale from 1, 2, or other factors may have resulted in a change in stool form at the time of determining the Bristol scale. 

The findings from this study highlight the substantial variability in the treatment type received for constipation in Japan. Most often, the treatment of constipation involves the use of laxatives and over-the-counter medication [48]. The mechanism of action of these agents can vary, and so can their efficacy and safety. In many cases, the adverse effects of many laxatives and over-the-counter medications have resulted in the use of natural food products as well [32,49]. Previous research indicated that in Japan, approximately 40% of individuals with self-reported constipation using laxatives used irritant laxatives [46]. Another recent study reported that magnesium laxatives are largely used in Japan [50]. However, there were many other different types of medication for constipation and combination treatments as well. Interestingly, a previous survey study found that treatment satisfaction for constipation symptoms decreased with an increased number of previously tried treatments [51], possibly due to increased symptom severity. Taken together, these findings may be useful for clinicians to assess and respond to each patient’s unique condition with the aim of better treatment, such as by conducting educational activities for those suffering from constipation. 

While constipation is a relatively common condition, chronic constipation poses a significant economic burden, as the cost of care is a significant concern for patients with constipation [29]. Data from the USA indicated that medical costs for the treatment of constipation ranged from USD 250 to 500 per year during the period of 1995–2003 [33]. Another study from the Netherlands showed similar medical costs of around EUR 300 during the period of 2006–2009 [52]. In Japan, the cost of constipation medication could range from JPY 1000 to 5000 (USD 10–50) [40]. The findings of this study suggest that more money spent on constipation treatment does not have a significant beneficial effect on QOL. Further analyses of the cost utility of constipation treatment should be performed. Previous work suggested that for customers, global IBS treatments tend to have the best cost utility [53]. A novel aspect of the current analysis was the focus on combinations of laxatives, which would impact cost. A future cost–utility analysis should consider different treatment types and costs. The finding that satisfaction was higher among those who spent less on treatment suggests that there may be financial inefficiency in the allocation of resources for constipation treatment. To the best of our knowledge, this is the first study to report a relationship between defecation satisfaction and treatment cost. While an increase in treatment cost did not necessarily result in better outcomes, it does not suggest that the cost of each medication is inversely proportional to treatment satisfaction. We cannot rule out the possibility that individuals spent a higher amount on treatment due to the use of several different laxatives (that may have been individually inexpensive) due to lack of efficacy. Our findings may have also been confounded by constipation severity. Patients with more severe constipation tend to spend more money on treatment and typically have worse outcomes owing to greater symptom severity. Further studies are required to examine this association longitudinally after controlling for baseline constipation severity.

The problem of chronic constipation has been shown to increase with increasing age [54], potentially due to changes in the chewing and swallowing ability of individuals as they get older [13]. One study found that the prevalence of constipation in individuals above the age of 65 was 16% in males and 26% in females. This prevalence increased by around 10% in both males and females above the age of 85 [55]. Given the increased size of the aging population in Japan, it is not surprising that the prevalence of constipation in Japan remains high. One study estimated that close to 30% of the population suffers from constipation [19]. Studies such as this will help identify potentially best practices for the treatment of constipation. It is important to treat constipation according to current treatment guidelines. Guidelines emphasize the following: accurate assessment and diagnosis of constipation type, laxative use, gastric emptying, and colonic and surgical interventions in extreme cases [29]. The findings of this study may help further define these guidelines and aid in the development of treatment guidelines specific to the Japanese population. In particular, for clinicians prescribing combinations of laxatives, certain combinations appeared to work better than others. For example, among the combinations used in this study, mean defecation satisfaction was highest for those who used a combination of irritant laxatives and saline, suggesting that this combination may be more effective than the others. 

This study has some limitations. Although the 3000 people included in the analysis were selected to ensure that the sample was representative of the Japanese population according to prefecture, they may not be representative of other factors, such as age and sex. Furthermore, the findings of this study were self-reported. The participants may have suffered from recall or other biases that may have influenced the findings. However, given that the constipation as well as defecation satisfaction data were self-reported, this study provides important information to help devise better patient-oriented treatment strategies. As a result of the constipation being self-reported, there was no way to distinguish between the type of constipation experienced by the individual, such as normal or slow-transit constipation. It is possible that the outcomes would have been different if the results were stratified according to constipation type. Additionally, data on the duration of constipation as well as the duration of treatment were not included in the survey, and hence it is unclear if and how the severity of constipation and the cost of treatment may have been influenced as a consequence. 

Despite these limitations, efforts must be made to address constipation in the Japanese population. Results from this study bring attention to the variety of constipation treatments used in Japan and show that defecation satisfaction is associated with improved BSFS scores and lower treatment costs. Further research should examine the optimal treatment course for patients with constipation. Additionally, research should aim to identify factors associated with a higher risk of constipation to enable the design of effective primary prevention strategies. 

## Figures and Tables

**Table 1:** Population demographics.

		n (%)	Age (+/−SD)	Taking Laxatives n (%)	Average Treatment Cost per Month in JPY and USD			
					Less than JPY 999 (USD 9.99)	JPY 1000–2999 (USD 10.00–29.99)	JPY 3000–4999 (USD 30.00–49.99)	JPY 5000 (USD 50.00) or More
Gender	Male	1503 (50.1%)	46.1 ± 13.4	432 (28.7%)	1107 (73.7%)	282 (18.7%)	75 (5.0%)	39 (2.6%)
	Female	1497 (49.9%)	46.2 ± 13.3	494 (33.0%)	1133 (75.7%)	286 (19.1%)	54 (3.6%)	24 (1.6%)
Severity of constipation (BSFS)	Bristol 1, 2	993 (33.1%)	45.6 ± 13.6	99 (47.8%)	686 (69.1%)	214 (21.6%)	56 (5.6%)	3 (3.73%)
	Bristol 3, 4, 5	1800 (60.0%)	46.3 ± 13.4	476 (26.4%)	1421 (78.9%)	299 (16.6%)	62 (3.4%)	18 (1.0%)
	Bristol 6, 7	207 (6.9%)	46.6 ± 12.9	352 (35.2%)	133 (64.3%)	55 (26.6%)	11 (5.3%)	8 (3.9%)

## Data Availability

The data presented in this study are available on request from the corresponding author. The data are not publicly available due to the Copyright Act.

## Supplementary Materials

The following supporting information can be downloaded at: https://www.mdpi.com/article/10.3390/jcm13113216/s1, Table S1: Questionnaire; Table S2: Association of IBS-QOL-J scores with variables [40,56,57,58,59,60,61,62,63].
