# Supplementary material for: Factors Associated with Defecation Satisfaction among Japanese Adults with Chronic Constipation
Source: J Clin Med. 2024 May 30;13(11):3216. doi: 10.3390/jcm13113216 (PMC11172833; doi:10.3390/jcm13113216)
Supplement: Supplementary file 1 [file jcm-13-03216-s001.zip › jcm-2909390-supplementary.pdf]

Supplementary Table S1: Questionnaire

Preliminary investigation

1. Please indicate your gender. (Male, Female)
2. Please indicate your age. (years old)
3. Please let us know the prefecture in which you currently reside.
4. Do you usually consider yourself constipated? If you are aware of constipation or constipation disorder, please indicate the degree of your constipation. (Very much agree, Fairly agree, Neither agree nor disagree, Not really agree, Not at all agree)
5. We would like to ask you a few questions. Are you currently pregnant? （Yes /No)
6. Have you ever been diagnosed or operated on by a physician for any of the following? Please answer "No" to all questions if you have never been diagnosed with a disease or had an abdominal surgery. (Yes, No)

1. hypertension, 2. diabetes mellitus, 3. hyperlipidemia, 4. inflammation of stomach and small intestine such as gastric ulcer duodenal ulcer, 5. inflammatory bowel disease/ulcerative colitis/Crohn's disease, 6. hemorrhoid disease, 7. diverticulitis/ diverticulosis, 8. cancer of digestive tract, 9. other cancer (Specify), 10. cerebrovascular disease/neurological disease such as cerebral infarction, 11. chronic obstructive pulmonary disease, 12. liver disease, 13. kidney disease, 14. other disease (Specify), 15. abdominal surgery other than appendicitis (including cesarean section surgery), 16. Other diseases (Specify:),

Detailed investigation

1. What is your height and weight? （   (cm)), ((kg))

2. We would like to ask you a few questions. Have you ever delivered a baby? （Yes/ No)

3. Do you take laxatives or laxatives? Please choose all that apply from the following. 1. stimulant laxative, 2. enema/suppository, 3. salt laxative, 4. other laxative or laxatives, (Specific name:), 5. do not remember the name but take laxative or laxatives, 6. do not take laxative or laxatives

4. Which of the following is the best way for you to obtain laxative or laxatives? Please answer for each of the following. (1. Buy laxatives at a pharmacy, 2. Ask your doctor to prescribe laxatives, 3. Buy laxatives online, 4. Other methods Specify:)

5. Next, how much money do you actually pay or are willing to pay for laxatives? Please choose one from the following. (1) 5０USD or more per month , (2)30USD to 49USD per month, (3) 10USD to 29USDper month, (4) 1 month 99usd or less.

6. Please answer the following questions regarding your current medication use. (1. Parkinson's medications, 2. epilepsy medications, 3. antidepressants, 4. medications for anxiety disorders or sleeping pills, 5. iron pills or iron supplements) (Other detailed survey items)

In addition to the above, questions on ROME III criteria [1], SF-8 [2], IBS-SI (Severity Index) [3], IBS-QOL [4], HADS (Hospital Anxiety and Depression Scale) [5], PSQI (Pittsburgh Sleep Quality Index (PSQI)[6], Multidimensional Perfectionism Scale (FMPS)[7], Japanese Health Practice Index (JHPI)[8], and Standard University Method (FODMAP)[9]. The total number of items was 67.

References:

[1] Drossman DA, Dumitrascu DL : Rome III : New standard for functional gastrointestinal disorders. J Gastrointestin Liver Dis 15 ; 237-241 : 2006

[2] Shunichi Fukuhara, Yoshimi Suzukamo: Health-related quality of life scale-SF-.8 and SF-36. in Igaku no Ayumi 213 ; 133-136 : 2005

[3] Saigo T, Tayama J, Hamaguchi T, et al : Gastroin-Testinal specific anxiety in irritable bowel syn- drome : validation of the Japanese version of the visceral sensitivity index for university students. Biopsychosoc Med 8 ; 10 : 2014

[4] Kanazawa M, Drossman DA, Shinozaki M, et al :.Translation and validation of a Japanese version of the irritable bowel syndrome-quality of life measure (IBS-QOL-J). Biopsychosoc Med 1 ; 6 : 2007

[5] Zigmond AS, Snaith RP : The hospital anxietyand depression scale. Acta Psychiatr Scand 67 ; 361-370 : 1983

[6] Farrahi Moghaddam J, Nakhaee N, Sheibani V, etal : Reliability and validity of Persian version ofthe Pittsburgh Sleep Quality Index (PSQI-P). Sleep Breath 16 ; 79-82 : 2012

[7] Frost RO, Marten P, Lahart C, et al : The dimensions of perfectionism. Cognit Ther Res 14 ; 449-.468 : 1990

[8] Yatabe H, Sugimori H, Suga M, et al: A study of the new Health Practice Index (JHPI) in Japan. Journal of the Japanese Society for General Health Examination 28 ; 302-310 : 2001

[9] Gibson PR, Shepherd SJ : Evidence-Based Dietary Management of Functional Gastrointestinal Symptoms : The FODMAP Approach. J Gastro- enterol Hepatol 25 ; 252-258 : 2010

Supplementary Table S2: Association of IBS-QOL-J scores with variables.

		**n (%)**	**IBS-QOL-J scores (mean± SD)**
Gender	Female	1497 (49.9)	80.1±19.9
	Male	1503 (50.1)	78.5±20.9
Academic background	Junior high school	78 (2.6)	79.3±21.0
	High school	907 (30.2)	79±20.5

	Technical collage	169 (5.6)	80.2±19.4
	Junior college	322 (10.7)	79.4±20.7
	Vocational school	193 (6.4)	78.4±20.1
	University	1173 (39.1)	79.3±20.6
	Graduate school	143 (4.8)	81±20.5
	Other	15 (0.5)	83.2±14.9
Annual Income	< 300 million yen (< $3000)	523 (17.4)	76.9±22.0
	300-400 million yen ($3000-4000)	392 (13.1)	80.5±19.0
	400-600 million yen ($4000-6000)	591 (19.7)	80.4±19.7
	600-800 million yen ($4000-6000)	404 (13.5)	79.7±19.8
	800-1000 million yen ($4000-6000)	285 (9.5)	78.7±21.1
	1000-1500 million yen ($10000-15000)	186 (6.2)	79.2±21.4
	1500-2000 million yen ($15000-20000)	60 (2.0)	80.0±19.6
	2000-2500 million yen ($20000-25000)	11 (0.3)	78.3±16.3
	>2500 million yen (> $25000)	18 (0.6)	73.1±20.5
	don't know	294 (9.8)	79.7±19.9
	don't like to answer	236 (7.9)	80.1±21.3
